# Barriers and Facilitators of Engaging Community Health Workers in Non-Communicable Disease (NCD) Prevention and Control in China: A Systematic Review (2006–2016)

**DOI:** 10.3390/ijerph15112378

**Published:** 2018-10-26

**Authors:** Hongfei Long, Wenting Huang, Pinpin Zheng, Jiang Li, Sha Tao, Shenglan Tang, Abu S. Abdullah

**Affiliations:** 1 Global Health Program, Duke Kunshan University, Kunshan 215347, Jiangsu, China; Hongfei.long@dukekunshan.edu.cn (H.L.); wenting@seshglobal.org (W.H.); Shenglan.tang@duke.edu (S.T.); 2 Department of Preventive Medicine, School of Public Health, Fudan University, Shanghai 200032, China; zpinpin@shmu.edu.cn (P.Z.); lijiang_fd@fudan.edu.cn (J.L.); taosha@fudan.edu.cn (S.T.); 3 Duke Global Health Institute, Duke University, Durham, NC 27710, USA; 4 Boston University School of Medicine, Boston Medical Center, Boston, MA 02118, USA

**Keywords:** community health workers, community-based intervention, non-communicable diseases, China, systematic review

## Abstract

*Background*: Non-communicable diseases (NCDs) have become a dominant disease burden in China. Although China has a prevention-centered NCD strategy, the implementation effect in the community has been subjected to manpower and financial difficulties. Engaging community health workers (CHWs) in community-based interventions may be a cost-effective approach to relieve the resource shortage and improve health. This review aimed to synthesize evidence on types of NCD-related care that was provided by CHWs in China, and to identify relevant barriers and facilitators. *Methods*: A literature search was conducted in Medline, PubMed, ProQuest, and Google Scholar databases for English-written, peer-reviewed articles published from 1996 to 2016 that reported findings from NCD-related interventions delivered by CHWs in China. Each article was extracted independently by two researchers. *Results*: Twenty distinct studies met the inclusion criteria. The two most common types of CHW-led NCD-related care were diabetes and hypertension management (*n* = 7) and mental health care (*n* = 7). Thirteen studies discussed the barriers and 16 studies reported facilitators. The most common barriers included lack of support (*n* = 6), lack of resources (*n* = 4), and heavy reliance on technology (*n* = 4). The common facilitators included an integrated health system (*n* = 9), community and patient trust (*n* = 5), high quality training (*n* = 5), and CHWs’ capacity (*n* = 5). Fourteen studies mentioned training content, while only eight described detailed procedures and duration. *Conclusions*: This review suggests that trained and supervised Chinese CHWs had the capacity to provide grassroots NCDs preventive interventions. In order to increase the generalizability and sustainability of such programs, studies with robust designs are needed to explore the effectiveness of CHW-led programs, and the intervention strategies to improve the practice of CHWs in various settings.

## 1. Introduction

In the latest decade, chronic non-communicable diseases (NCDs) have become a dominant disease burden. In 2005 in China, 80% of deaths and 70% of disability-adjusted life-years lost (DALYs) were attributed to NCDs [1,2]. Many people suffering from NCDs remain untreated due to inadequate attention and funds from the governments [3]. Meanwhile, as China has seen economic growth with its population aging, lifestyle changes, and socioeconomic transition, the prevalence and impact of this NCD epidemic are likely to expand over time [4].

To curb this epidemic, China has implemented a series of nationwide community-based NCD prevention and control programs, including hypertension screening, counseling, salt intake reduction education, healthy lifestyle promotion, and smoking cessation [5]. Most of these programs have been delivered to community members by different categories of public health workers at primary care level (e.g., doctors and nurses in county-level clinics and Centers for Disease Control). However, there are still needs to mobilize other sustainable human resources at community level, especially in remote rural areas, to reach the public with wide categories of evidence-based NCD prevention and control efforts.

Evidence shows that engaging community health workers (CHWs) in community-based health care programs can relieve the health professional shortage and improve the population health in a cost-saving manner [6]. The World Health Organization (WHO) defines CHWs as the public health workers who are selected by community members, and who live in the communities they serve [7]. However, as CHWs are given different names from their assigned tasks associated with local cultures and health systems (e.g., traditional birth attendants, community health volunteers, village health workers, etc.), there is no any universal title for all CHWs [8]. For example, in China, CHWs were initially named “barefoot doctors” from the 1950s to 1980s [8]. Since then, in the 1981 state healthcare reform, the barefoot doctors have been entitled as “village doctors”, if they passed the national qualification exam after receiving basic medical training [9]. Usually, village doctors receive three years of junior college education and one year of in-service training in a medical practice. They also have to receive continuing education of 15 credit hours (accounting for 45–90 class hours) per year. We will use the term “CHWs” to describe all these categories of healthcare workers in this paper, unless specified otherwise.

In recent decades, Chinese CHWs have been actively engaging themselves in NCD prevention and control services. According to the China Ministry of Health, NCD-related community-based health services should include elderly health management, case management for hypertension, type II diabetes, and mental illnesses [10]. Recent policy reforms have shown a strong interest in shifting chronic disease management tasks to village doctors [11]. Moreover, the literature has revealed that with proper training, support, and supervision, CHWs can deliver services such as diabetes screenings, cardiovascular diseases case management, healthy lifestyle counseling, early detection, referral for priority chronic diseases, and medication compliance management in NCD programs [12,13,14,15]. However, these have not been officially included in the CHWs’ responsibilities and agendas.

Given the huge potential for CHWs in their contributions to NCD prevention and control in China, there is a need to generate an efficient and feasible strategy to strengthen their roles in addressing NCDs in an organized manner. Nevertheless, there has been limited literature that summarized the range of NCD programs led by CHWs in China, or that examined the challenges and facilitators encountered by CHWs when delivering NCD-related preventive services. As China is working to implement the Medium-to-Long Term Plan for the Prevention and Treatment of Chronic Diseases (2017–2025), the findings from this systematic review can and will inform policy and public health interventions surrounding community health workers in China. Therefore, the purposes of this review were as follows: (1) to document the types of NCD-related healthcare or preventive services that were delivered by CHWs in China; and (2) to summarize the barriers and facilitators for engaging CHWs in NCDs prevention and control at primary care level in China. This systematic review will be guided by the following research question: What are the types of NCD related health programs provided by CHWs in China as reported in studies from 1996 to 2016, and what are the barriers and facilitating factors?

## 2. Methodology

### 2.1. Data Sources

This systematic review investigated all the English language, peer-reviewed journal publications that entirely or partially focused on the roles of CHWs in common NCD prevention and control programs in China.

We started this review by searching the published journal articles on Medline, ProQuest, Google Scholar, and Scopus, using text words ‘community health workers’, ‘village doctors’, ‘non-communicable diseases (NCDs)’, and ‘China’, as well as corresponding medical subject headings (MeSHs) terms. We also searched for non-MeSH terms such as ‘lay health workers’, ‘lay health supporters’, ’community health promoters’, ‘community’, ‘rural’, and ‘chronic’, as well as their synonyms in English to expand the inclusion of available studies. Meanwhile, we also checked the reference lists of the identified articles to explore additional publications to broaden the inclusion criteria.

### 2.2. Study Selection

We targeted articles that were written in the English language, with interventions conducted in China and published in the last 20 years (1996–2016). We used the publication date instead of the study date, as the publication date is relatively more accessible.

The inclusion and exclusion criteria for CHWs-delivered studies followed the PICOS framework [16] as follows:

1. Participants: Participants can be patients suffering from the aforementioned NCDs, NCD high-risk populations, or the general populations without restricting types of participants, because preventive services and interventions can reach different populations based on specific needs and settings.
2. Intervention types: NCD-related preventive measures or health promotion interventions that were provided by CHWs, village doctors, lay health supporters, or those healthcare personnel who delivered community-based services but received less formal training than health professionals.
3. Comparison: Not applicable.
4. Outcome: Delivery of reported intervention, provider types, categories of NCD topics, and challenges and facilitators.
5. Study types: Both descriptive and evaluation studies conducted in China focusing on community-based NCD-related programs (including programs focusing on the chronic diseases as well as the NCD risk factors), such as hypertension screening, physical activity promotions, early detection for cardiovascular diseases (CVDs), diabetes, and mental health illness.

The exclusion criteria included the following:

1. Articles that did not focus on China.
2. Articles that focused on the health professionals (physicians, doctors, nurses) rather than CHWs as we have defined for this review.
3. Articles that mainly discussed general primary health care (i.e., non-NCD related services), such as immunization, infectious disease case management, family planning, reproductive health, environmental health, and maternal and child health.
4. Articles that did not describe structured NCD-related interventions (e.g., news, conference reports, books, reviews, health system analysis, disease prevalence).

**Figure 1:**
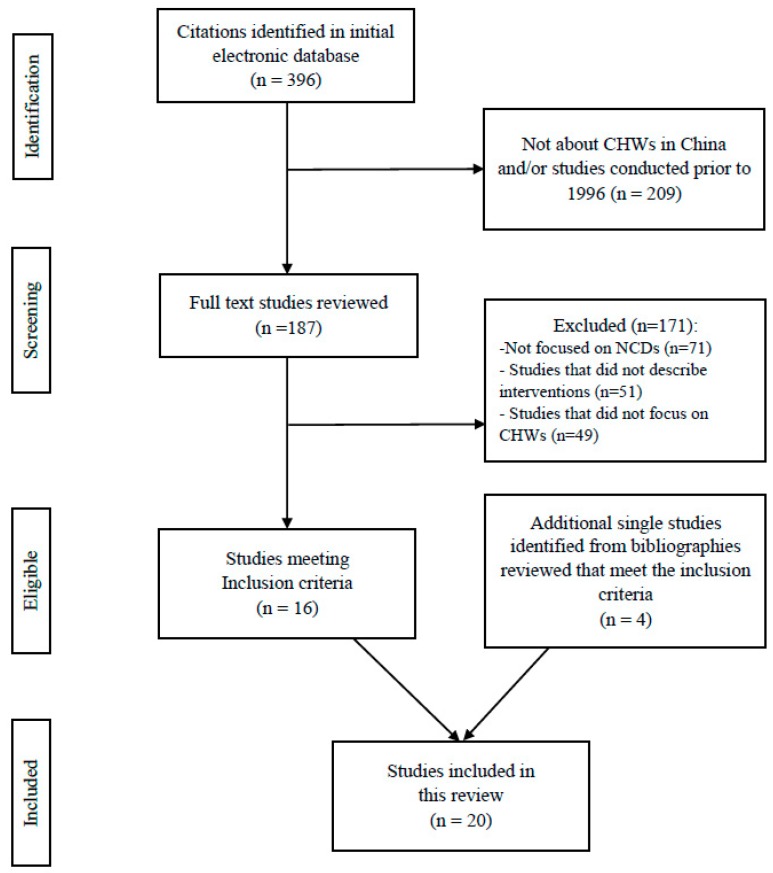
Selection process for identifying relevant studies. NCDs, non-communicable diseases; CHWs, community health workers.

Two members of our research team (W.H. and H.L.) independently assessed the relevant studies based on the inclusion and exclusion criteria. Each reviewer scanned the titles and abstracts of all potential articles to determine eligibilities. Disagreements were resolved consensually, first by these two members and in some cases with inputs from the project leader (A.S.A.). The study selection process in this review followed the PRISMA flow diagram [17] and is summarized in Figure 1.

We then read the full texts of all eligible materials and summarized relevant content. Using an Excel form, we assigned each eligible article with a unique reference number and extracted the following information: the types of program, titles of CHWs, the services provided by or/and the responsibilities of CHWs, program duration, training received by CHWs, and challenges and facilitators faced by CHWs in the engaged program. We also summarized the training types received by CHWs as well as the training duration, if this information was available (Table 1).

## 3. Results

### 3.1. General Description

The literature search yielded 396 potentially relevant articles. After screening for eligibility and reviewing inclusion and exclusion criteria, twenty studies were retained (Figure 1). As shown in Table 1, of these twenty studies [13,14,15,18,19,20,21,22,23,24,25,26,27,28,29,30,31,32,33], eleven studies were single-site programs [13,14,18,19,20,24,25,26,28,29,30,31]; nine studies discussed multi-site programs [15,21,22,23,27,31,32,33,34]; while two of them did not mention specific locations of the study sites [22,25]. Among the programs with specific study sites (*n* = 18), the majority were located in the east and central parts of China (*n* = 11) [14,18,20,21,23,24,25,26,27,28,31], followed by western (*n* = 5) [15,30,32,33,34], northern (*n* = 1) [19], and southern China (*n* = 1) [29].

Most programs included in this review lasted over one year [13,14,18,19,23,24,25,26,27,28,29], while a few lasted five years or more [14,18,29]. Five studies were on-going programs [13,14,18,29,31].

The title of the CHWs varied in different programs. Ten programs named the CHWs as ‘village doctors’ [14,18,19,20,22,26,30,31,33,34], while the other ten programs [13,15,21,23,24,25,27,28,29,32] used ‘health coach’, ‘local health workers’, ‘CHWs’, ‘lay family health promoters’, ‘community health service center staff’, and ‘primary health care providers’.

### 3.2. NCD-Related Services Provided by CHWs

NCD-services provided by CHWs are categorized in five groups: (1) diabetes and/or hypertension (seven studies) [13,18,19,20,21,22,23]; (2) cancer (two studies) [14,24]; (3) mental health (seven studies) [25,26,27,28,29,30,31]; (4) CVDs (three studies) [15,32,33]; and (5) NCD risk factors (one study) [34].

#### 3.2.1. Diabetes and Hypertension

All seven papers that focused on diabetes and hypertension, case management, prevention, and monitoring were the major responsibilities of CHWs. Outreach services such as follow-up via home visits [13,18,19,20,21] and behavioral change counseling on healthy diet, physical activities, mental health, and high-risk behavior prevention [13,18,20,21] were largely provided by CHWs in these studies. In two studies, CHWs promoted and conducted screening, or assisted in early detection of high-risk populations [18,20].

#### 3.3.2. Cancer

In the program developed by Belinson et al. [24] for community-based cervical cancer screening, community leaders and CHWs worked together to help health professionals gather personal information and promoted the program via video, posters, and educational workshops within the communities. Another on-going study conducted by Chai et al. [14] introduced a systematic approach in cancer prevention by engaging village doctors.

#### 3.2.3. Mental Health

Among the seven studies under the mental health category, four focused on schizophrenia [26,27,28,31], one on dementia [25], one on late-life depression [30], and one on general mental illness [29]. 

#### 3.2.4. Cardiovascular Diseases (CVDs)

The CHWs in all the three CVDs programs [15,32,33] shared similar responsibilities: identifying high-risk patients, providing lifestyle modification recommendations, measuring blood pressure, and referring patients to higher-level clinics.

#### 3.2.5. NCD Risk Factors

Only one study described the NCD risk factors [34]. In this large-scale salt reduction intervention, village doctors distributed promotional materials and salt substitutes, and conducted interactive education for people with a high risk of vascular diseases.

### 3.3. Training Received by CHWs

Fourteen studies had at least some description of the training content received by CHWs [13,15,18,20,22,23,24,26,29,30,31,32,33], among which eight studies [13,14,15,24,26,30,32,33] included detailed information about the training procedures and training duration. Training contents reported was program-specific [15,32,33], while training types included web-based training [14,18,20,31] and workshops [13,24,30].

### 3.4. Barriers

Thirteen studies discussed different barriers of engaging CHWs in NCD prevention and control programs [13,14,15,18,19,20,21,22,23,26,29,30,31,32], as summarized below:

#### 3.4.1. Lack of Support from the Local Health Systems and from the Public

Inadequate support from the health system and policy was one of the major barriers in four articles [19,22,26,29]. Particularly, Li et al. [22] stated that some rural villages were not covered by the New Cooperative Medical Scheme; therefore, the local CHWs did not have official contracts, which caused dissatisfaction among the CHWs. A lack of support from patients also reduced CHWs’ job motivations [20,26,29,30].

#### 3.4.2. Lack of Resources

Ajay et al. [32] and Tian et al. [15] pointed out that limited economic and health care resources in the poor areas were the major barriers. Xu et al. [31] reported that inadequate local psychiatrists compromised the village doctors’ performance, as they did not have professional back-up to manage difficult cases.

#### 3.4.3. Heavy Workload

Three papers reported that heavy workload with the already assigned services was a barrier for CHWs to provide NCD-related services [18,26,29]. The village doctors who were already busy as general primary health care providers were reluctant to increase their workload by adding extra NCD tasks [18,26,29].

#### 3.4.4. Inadequate Financial Incentives

In three programs, CHWs were not fully satisfied with the financial incentives they had been receiving [22,29,30]. In these studies, their current wage was lower than their expected wages.

#### 3.4.5. Heavy Reliance on Electronic Technology

Several studies discussed problems associated with the increasing reliance on technology in the intervention delivery by CHWs [14,18,20,23]. First, Chen et al. [20] and Peiris et al. [23] claimed that even smartphones service was still not accessible for regions with extremely poor conditions. Second, Feng et al. [18] and Chai et al. [14] indicated that the computerized system operation might go beyond the capability of village doctors in remote areas.

#### 3.4.6. Lack of Knowledge and Skills

Three studies reported the inadequate knowledge and skills of CHWs as a barrier [18,29,30]. In a CHW-led diabetes prevention program conducted by Feng et al. [18], most village doctors were unaware of the importance of diabetes prevention. In another paper by Ma et al. [29], CHWs’ lack of professional mental health expertise limited their capabilities of providing relevant services. One study [30] revealed the unsatisfying training quality by highlighting the limited time for practice.

### 3.5. Facilitators

Sixteen studies [13,14,15,18,19,20,21,22,23,24,26,29,30,31,32,33] indicated at least one facilitator for CHWs to provide NCD prevention and control services. The following categories present a summary of the enabling factors identified:

#### 3.5.1. Integrated Health System

Nine studies mentioned that a close collaboration among policy makers, high-level public health institutes, and CHWs was beneficial [18,19,22,23,24,26,30,31,32]. In Feng et al. [18], the village clinics prepared appropriate settings and resources to make it convenient for CHWs to measure blood pressure and conduct counseling. In another diabetes management program [22], village doctors were better managed when they had access to contracts to the New Cooperative Medical Scheme.

#### 3.5.2. Community and Patient Trust

Trust from patients and communities was another facilitating factor [12,16,18,26,27]. Particularly in the studies of Feng et al. [18] and Xu et al. [31], mutual trust between CHWs and patients facilitated smooth delivery of CHWs’ work, and lead to better medication adherence and patients’ satisfaction.

#### 3.5.3. Incentives

Although no study mentioned that monetary incentives could engage CHWs in the delivery of NCD programs, the village doctors in one hypertension and diabetes control program confessed that they hoped to receive additional subsidy [22]. Moreover, performance-based incentives were considered efficient in increasing CHWs’ job motivations and improving their work performance [14,15,18,33].

#### 3.5.4. Appropriate Training

In multiple NCD programs, the trainings were designed to meet CHWs’ competences and expectations, so that CHWs were able to quickly understand key concepts and acquire skills [15,18,22,26,30].

#### 3.5.5. Capacity of CHWs

Five articles [13,21,24,29,30] included CHWs’ capacity (i.e., competencies and communication skills) to deliver the required services as a positive influencing factor. Browning et al. [13] observed that CHWs with better learning capacity could quickly transform the knowledge and skills into practice. Belinson et al. [24] and Ma et al. [29] found that CHWs who possessed high communication skills could effectively assist patients in changing unhealthy behaviors.

#### 3.5.6. Interests and Attitudes

Three studies discussed CHW’s interests and attitudes as facilitators. Belinson et al. [24] and Tang et al. [30] stated that CHWs were strongly enthusiastic about providing related services in their studies on promoting cervical cancer screening and conducting cognitive behavioral therapy intervention. Another study about mental health care conducted by Ma et al. [29] reported that CHWs without discrimination towards mental illness could more effectively communicate with patients and provide appropriate care.

## 4. Discussion

This review provided contemporary evidence of the roles that CHWs could serve in preventing and controlling NCDs in China, and the corresponding barriers and enablers. Despite variations in the study settings, methods, and program durations of the identified papers in this review, the findings indicated that Chinese CHWs provided certain types of community-based preventive services on major chronic diseases. These were generally aligned with the results of other international reviews [35,36,37,38]. However, limited studies in this review focused on NCD risk factors management among general populations, except one sodium reduction program conducted by Li et al. [34]. Common preventable NCD risk factors should not be neglected in community-based NCD programs [39]. Studies in other low- and middle-income countries (LMICs) introduced CHWs’ roles in promoting tobacco control, physical activities, and healthy diets among non-patients [40,41,42,43]. One program conducted by Reininger et al. [41] integrated CHWs’ home visits with the community campaign to educate the Mexican descent population about the significance of physical activity and healthy food consumption in reducing NCDs. The results showed that the participants who received both CHW meetings and radio messages were likely to increase physical activity, and the participants who were exposed to the program were likely to consume more fruit and vegetables than those who were not. These studies underscore the potential to engage CHWs in the delivery of NCDs risk factor reduction intervention; however, further studies on CHWs’ feasibility in providing such services in China are needed.

The barriers that we identified in the current study (i.e., inadequate knowledge and skills; heavy workload; limited resources and support) are consistent with the findings in other local and international studies [22,37,44,45]. Consistent with other studies [46,47,48,49,50], the insufficient knowledge and skills of CHWs, as was identified in the current study, could possibly be resolved by delivering organized training that will strengthen CHW’s skills and promote positive attitudes towards NCD care at the community level.

The lack of support from the health system was identified as a key challenge in this review. While evidence from the identified articles [18,19,22,23,24,26,30,31,32] and previous studies [51,52,53] also indicated that the support from integrated management and local collaboration would lead to effective CHW-led interventions and assist in scaling-up programs. The integrated management strategy has been enacted since the 2009 health sector reform in the rural health care service scheme. It requires local township health centers (THCs) to take charge of village clinic management, including personnel, finance, facilities, medicine, and all works associated with village doctors [53]. Rural CHWs can seek training, as well as financial and facility support through the contracts with THCs [54]. However, limited to local economic and government interests, not all the CHWs can acquire the support they need.

One unexpected finding in this review was that only three identified papers listed low individual financial incentives as a barrier, while other domestic and international studies included it as a major challenge for CHWs’ engagement [22,45,55,56]. Chinese CHWs are paid by the central and local governments [52,53], thus it seems that they have relatively less economic difficulties compared with the voluntary or informally-paid CHWs in other countries. However, in fact, researchers have revealed that multiple income disparities exist among CHWs in China. First, although the government pays one part of the CHWs subsidy based on national standard, the delivery of the other parts of the subsidy managed by local THCs vary largely in different settings and are subjected to the local fiscal ability [57]. In addition, despite that local THCs should pay no less than 40% of the government funding to the village clinics, the THC supervisors sometimes delayed or withheld the funds as a punishment strategy for those village doctors who did not accomplish all the assigned tasks [58]. Furthermore, the New Cooperative Medical Scheme (NCMS) covers healthcare at THCs and county-level hospitals, while it does not cover all village clinics. Patients covered by NCMS would prefer going to the THCs and county-level hospitals rather than the village clinics [59]. As a result, the village doctors would lose the opportunities to both receive subsidy from the policy and provide needed care to the patients. The findings in this review [14,15,18,33] suggested that performance-based incentives were widely used as an income strategy to motivate and engage CHWs in the delivery of NCD-related health interventions, although the large-scale validity is uncertain. Therefore, future studies and policies need to be concerned about how to expand the coverage of NCMS contract to all village clinics, how to allocate funding and limited resources to better remunerate and motivate CHWs, and how to ensure commitments to human resources development and capacity building [6,60,61,62].

We found that several factors (i.e., receiving training, competencies, and skills to deliver NCD-related services, as well as community trust) encouraged CHWs to engage in the delivery of NCD-related services. While we argue that community trust towards CHWs could be affected by the local culture in receiving healthcare services from non-medical professionals, and whether CHWs are well known within the community, measures need to be taken to improve CHWs’ capacity as well as trust within the community.

There are several limitations of this study. First, this review excluded many studies considering their absence of information on CHWs’ training, services provided, and/or influencing factors that could not meet our inclusion criteria. This information shortage itself could indicate a gap in the current research field that Chinese CHWs’ roles as formal ordinary healthcare providers in NCD prevention and control are still neglected, thus further studies to comprehensively explore their potential capability in NCD care provision are needed. Second, this review did not focus on effectiveness of the selected studies. As the aim of the study is to explain the current role and the feasibility of Chinese CHWs, whether those programs including CHWs would have an efficient outcome was not further explored. Therefore, we included some on-going programs that did not offer any evaluation. Further follow-up investigations on the evaluation of those on-going studies are needed in order to assess the contribution of getting CHWs involved in these programs. Third, despite that most identified studies in this review showed that involving CHWs in the programs would likely generate positive program outcomes, there were limitations on the generalizability of those findings. For instance, the program successfully conducted in the urban sites would not be replicable for the population in rural settings because of socio-cultural differences, socio-economic constraints, and study design variations. Also, a comprehensive cost-effectiveness analysis was not mentioned or planned in any of the identified studies. The measures and approaches taken for intervention effectiveness evaluation varied greatly across programs. Future studies with rigorous study designs among larger populations are needed to clarify the generalizability of the findings and to establish more appropriate models in planning, implementing, and evaluating large-scale CHW-led NCD intervention programs. Also, applicability of such programs in both rural and urban capabilities and expectations would be needed. Systematic efforts from and close communication between the central and local stakeholders are warranted during the entire process. Finally, as a result of resource constraints, this review only included English-written studies. In the next stage, a systematic review of the Chinese literature would be integrated as one critical supplementary part to the current study.

## 5. Conclusions

Overall, this review has provided insights into the pattern of NCD-related health interventions that are provided by CHWs in China, and summarized potential barriers and facilitating factors. The findings suggest that Chinese CHWs, upon receiving training, are capable of delivering non-invasive health care services for patients with chronic diseases; however, CHWs’ engagement in the delivery of NCD risk factor reduction interventions for general population was not common. The barriers and facilitators identified ranged from personal level to health system level factors that could influence CHWs’ working motivation and performance. These factors should be taken into account by policy makers and other stakeholders to develop strategies for effectively using the workforce of CHWs in the grass-root NCD prevention and control program implementations.

## Figures and Tables

**Table 1:** Description of non-communicable disease (NCD)-related studies and programs that engaged community health workers (CHWs) in China.

No.	Author	Year; Location	Type of NCDs	Provider Type	Payment Mechanism/Structure (Paid/Unpaid)	Indicators							Key Findings/Lessons Learned
						Types of Services	Program Duration	Ended/Current	Types of Training	Training Duration	Challenges (−)	Facilitated Factors (+)	
**Diabetes and/or hypertension**													
1	Feng et al. [18]	2013; Lu’An, An’hui Province	T2DM	VDs	paid	1. Glucose screening; 2. measured body weight and blood pressure; 3. counseling on glucose screening; 4. promoted screening participation via biannual follow-up glucose screening; 5. referral; 6. behavioral change counseling for pre-diabetics	6 years (every 12 months for plasma glucose and ever month for body weight and blood pressure)	Current	Web-based training, with an ‘occupational toolkit’ consisting of a workbook, a manual guidance, and a set of cue-cards. For example, each cue-card enlisted critical steps or elements for delivering a specific type of counseling; the manual was a reference book including elementary protocols on common problems and solution tips for diabetes prevention	N/A	1. Most VDs were unaware of diabetes prevention; 2. already heavy workload; 3. heavy reliance on electronic/commuter support may beyond the ability of VDs’ and elder villagers’ in rural areas	1. Trust from patients and communities; 2. service was simple, capable for VDs (only 15 minutes); 3. well-established guidelines and manuals; 4. village clinics was appropriate setting; 5. electronic support and web-based training were cost-saving and time flexible; 6. performance-based incentives; 7. local health authorities support on resources	The study examined key success factors in a synergistic way towards integrated and sustainable diabetes prevention. It introduced a package of long-term incentives and continuous capacity building mechanism. It built up a potential operational cycle for catalyzing similar efforts in the local prefecture
2	Lin et al. [19]	2014;Xilingol county; Inner Mongolia	Hypertension; Diabetes	VDs	paid	1. Case management and monitoring via EHR; 2. regular followed-up check on medication compliance; 3. measured blood pressure and blood sugar levels	2008–2011	Ended	N/A	N/A	Lack of policy support from the health system	Close connection with higher levels of the healthcare system, if implemented in large-scale	Cloud-based EHR had the potential to improve care provision in rural China and efficiency of disease management.
3	Chen et al. [20]	2014; Lu An; Anhui Province	T2DM	VDs	paid	1. Identified high-risk patients; 2. case follow-up counseling and health education on diabetes risks, balanced diets, and physical activities	6 months; 1 month/session	Ended	Instructions on the application method of the program, with standardized “step-by-step” navigation for VDs to follow in practice	N/A	1. Lack of electricity security (facility) in remote settings; 2. communication difficulties: sometimes unable to engage patients to finish all the required interventions	1. Easy to follow the navigation; 2. professional knowledge built in the application helps case identification and management; 3. high acceptance rate among patients	The web-based tool SWAP-DM2 can increase the effectiveness of T2DM preventive services delivered by VDs, and may increase the frontier clinicians’ participation in diabetes prevention in similar settings.
4	Zhong et al. [21]	2015; Tonglin, Hefei, Bangbu, Anhui Province	Diabetes	Peer Leaders; CHSC Staff	N/A	Bi-weekly educational meetings co-led by PLs and CHS staffs. Topics: diets, physical activities, medications, foot care, stress management. PLs’ responsibilities: outreach, promotions, emotional support, and non-professional activities (Tai Chi, morning exercise, etc.)	6 months/session	N/A	N/A	N/A	Lack of staff resources in sub-communities	1. Close relationship with peer leaders; 2. knowledge; 3. high patient engagement	The PLSP was effective for subcommunity-based diabetes self-management. May be generalizable to cardiovascular prevention.
5	Li et al. [22]	2015; three provinces in China, specific location not mentioned	Hypertension; Diabetes	village doctor	paid	1. Hypertension and/or diabetes case management; 2. created citizen health record (no specific information provided for this category)	2014 (cross-sectional survey among VDs)	Ended	Routine training programs including content such as health care policy; standards; BPHS quality management; and the norms, standards, and service delivery paths of BPHS.	N/A	1. Limited compensation and low financial incentive; 2. Uneven geographic coverage of the NCMS insurance contract	1. Education and training opportunities, 2. public health care subsidy; 3. integrated management and supervision; 4. access to NCMS insurance program contract	Increasing subsidies for VDs, availability and attendance of training opportunities, integrated management, and NCMS contracting of village clinics were important factors in increasing BPHS provision in rural areas.
6	Browning et al. [13]	2016; Fengtai District, Beijing	T2DM	Health coach (health workers from the local CHS)	paid	Bi-weekly/monthly telephone and face-to-face motivational interviews (MIs), to provide psychosocial support and lifestyle counseling to improve the outcome of glycemic control and self-care of T2DM patients.	1 year	Current	Key concepts in patient-centered communications, health psychology, epidemiology of targeted conditions, the framework and rationale of MIs, and the application of MIs core skills. Review workshops were arranged one month after the project initiation, and every three months after that.	Initial training: 20 hours; review workshop: half day per workshop	Not generalizable to rural settings with limited human resources	1. Good learning and practice capacity; 2. well-organized training process including review workshops	The result of this study indicated that the coach-led psychological intervention had the potential to improve mental health status and well-being of T2DM patients. The intervention could provide evidence for the establishment of regular and free clinical health checks for people with T2DM.
7	Peiris et al. [23]	2016; Beijing; Hebei Province	T2DM	FHPs; Healthcare staff	Healthcare Staff: Paid; FHP: unpaid	Healthcare workers: case monitoring, provided support to FHPs via communication tools built inside the mHealth application; FHPs: reported the progress and update EHR data on behalf of the patients (i.e., their family members who have diabetes) via the mHealth application. Co-determined action plan with the support from healthcare workers. Experience sharing with other FHPs in the community via application-based forum.	2 years: 2016–2017	N/A	Installation and the use of the technology and management of diabetes	N/A	Findings were not generalizable for other contexts without EHR infrastructure, and for the population with limited access to smart-phone technology	1. Cost-saving; 2. time-saving; 3. strong motivation of FHPs to support families with diabetes; 4. close communication between clinical healthcare staffs and FHPs.	With FHPs’ and community healthcare staff engagement, the application-based mHealth intervention had the potential to increase the quality of treatment among diabetes patients, and to scale-up the intervention in similar settings.
*Cancer*													
8	Belinson et al. [24]	2014; Henan Province	Cervical Cancer	CLs; Promoters;local health worker	paid	Joint tasks for CLs and promoters: gathering personal information and labeling specimens; following the procedures; promoting screening via video, posters, workshops. CLs: instruct sample collection. Local health workers: consultations and referrals after results generation.	2011–2013	Ended	Meaning of a positive test; management options and techniques; via video and workshops	1 day	N/A	1. Good communication skills; 2. enthusiasm for the job; 3. community, institutional, and government support	This community-based self-sampling model was capable of developing massive screenings. Improvements can be made when local doctors are trained to manage the positives.
9	Chai et al. [14]	2015; An’hui Province	cancer	VDs	paid	1. Health counseling regarding alerting risks and harms, discussing efficacy and benefits, and anticipating barriers; 2. risk assessment promotions; 3. support on healthy lifestyle behavioral changes (reviewing behavior changes, encouraging improvements, identifying and solving problems); 4. manage, record, and upload typical cases bi-monthly on a web forum and shared experiences with other experts and VDs	5 years	On-going	Web-based tutorial on implementing the project prevention in both video and textual formats; typical case studies as references for practice; video and pictorial materials about cancer and its prevention	One-day orientation workshop	Heavy reliance on electronic support, so the actual practice may beyond the ability of VDs in remote rural areas to use computerized systems	1. Performance-based incentive and awards; 2. web-based support and supervision system were time-saving; 3. user-friendly education and learning assistance; 4. self-practice, encouragement, problem inquiry, and answering in the training allowed most village doctors to become confident users of the electronic support system	This international program was to empower advocacy, raise awareness about dementia, and ensure that the health and social care needs of older people were met in low- and middle- income countries
**Mental Health**													
10	Prince et al. [25]	2007; urban and rural catchment, no specific location mentioned	dementia	CHWs	paid	1. Help researchers to detect high-risk populations; 2. being the community key informants of the research team	2 years	Ended	N/A	N/A	N/A	N/A	Village workers with non-clinical training have the potential to manage severe psychiatric diseases and other chronic conditions.
11	Gong et al. [26]	2014; Liuyang, Hunan Province	schizophrenia	VDs	paid	1. Manage case files for patients; 2. store and distribute antipsychotics to family members on a weekly basis, or directly observe drug-taking at the village clinic on a daily basis; 3. accompany patients and family members on bi-monthly visits to psychiatrists for drug dispensation; 4. record patients’ medication-taking behavior weekly; 5. identify signs of relapse; 6. referral.	1 year	Ended	Mental health knowledge, case-management skills, and DOT.	3 days	1. Already overload, no time for extra work; 2. no financial compensation for extra efforts; 3. inadequate engagement from patients and patients’ families	1. Under the national “686” mental health scheme—government support; 2. consistent collaboration with local government; 3. training met local VDs’ competence and expectations	The results of this experiment provided evidence on the role of health workers with relatively limited medical training in managing severe psychiatric disease and other chronic conditions in developing countries.
12	Chen et al. [27]	2014; Xuhui and Hongkou Districts; Shanghai	schizophrenia	CHWs	paid	Worked with community psychiatrists and nurses as a team to conduct case management: 1. Assess recovery status, employment status, and social activities of patients; 2. assist patients to develop personalized rehabilitation plan and cope with the plan, drug adherence training, daily skills training, and family psychological intervention; 3. monthly follow-up to refine the plan	2 year	Ended	N/A	N/A	N/A	N/A	The study highlighted the need to involve family members in the management of patients’ medications, to use the minimum effective dosage of medications, and to manage all side effects.
13	Zhou et al. [28]	2014; Shanghai	schizophrenia	CHWs	paid	Assist patients with self-management. After each patient received weekly self-management skill training, CHWs reviewed patients’ self-management checklist (recorded their daily adherence quality of sleep, occurrence of side effects, occurrence of residual symptoms and early signs of relapse, daily activities, and general mood) every month at a group meeting to supervise the adherence and collect record	2.5 year	Ended	N/A	N/A	N/A	N/A	Self-management training could introduce a reduction in relapse and improvements in chronic schizophrenia medication adherence. Cost-benefit studies are needed to assess the feasibility of up-scaling this intervention.
14	Ma et al. [29]	2015; Guangxi Province	mental illness	primary health care providers	paid	1. Community education; 2. medication distribution; 3. observed compliance and life status; 4. report side effects or any abnormality; 5. referral and follow-up	2006–present	Current	Training provided by the national ‘686 project’: mental health disease management, education and social treatment, and prevention of mental illnesses	N/A	1. Lack of professional knowledge; 2. fear of patients’ attack; 3. extra workload; 4. no available management approach; 5. insufficient subsidies	1. Communication skills; 2. proper attitudes (without discrimination); 3. improved knowledge; 4. increased income/subsidy	Improvements can be made regarding 1. trainings on professional mental health knowledge and attitudes; 2. management approach; 3. income/subsidy
15	Tang et al. [30]	2015; Mianzhu, Sichuan province	late-life depression	VDs	not paid	Conducted weekly intervention with elderly depression patients using CBT techniques to 1. perform physical examinations; 2. identify emotion status and negative thoughts; 3. proceed psychological interventions; 4. provide problem solving methods	2 months	Current	Workshops on mental disorder knowledge, counseling concepts, and techniques, with specific focus on CBT. Practice through role-play. Trainings were conducted by a qualified cognitive therapist	Six full days (three consecutive weekends)	1. Time constraint for training; 2. under-developed training manuals and inadequate practice 3. poor patient adherence—preferred medicine over CBT; 4. no financial incentive	1. Well designed (easy to understand the content) and organized (the use of role play) training; 2. strong learning ability and interest; 3. local community trust; 4. multi-disciplinary team	The study highlighted the feasibility and good patient acceptability of including CBT in the treatment process for the rural elderly. Remaining challenges: 1. A lack of step-by-step treatment manuals in the training; 2. a lack of support from family; 3. a need to integrate CBT intervention into public health services.
16	Xu et al. [31]	2016; Liuyang, Hunan Province	schizophrenia	VDs; LHS: mostly family members of the patients	VD: paid; LHS: unpaid	VDs: 1. Screening, as the “686” scheme requires; 2. reported relapse signs and side effects to psychiatrists; 3. teamed up with LHSs, MHA, and psychiatrists to assist urgent care. LHSs: 1. Facilitated patient medication adherence with prompts from the e-reminders; 2. monitored for early signs of relapse and side effects using checklists from the e-monitor and report to VDs; 3. teamed up with the VDs and the township MHA to facilitate treatment adjustments and urgent care	1 year	On-going	The built-in e-educator mHealth program sent periodic SMS messages to patients, LHSs, MHA, and VDs to educate them on schizophrenia symptoms, medication, adherence strategies, relapse, rehabilitation, and social resources	N/A	1. Local psychiatrists with limited training may deliver inappropriate services; 2. funding for the program may not be sustainable in the future.	1. Policy support; 2. individual and community engagement (MHAs, psychiatrists, VDs, patients and their families); VDs: No additional workload; LHSs: 1. Care and love for their families (i.e., patients) as the major job motivation; 2. non-monetary reward.	The design of the LEAN program was expected to be a cost-saving, feasible, and generalizable community-based schizophrenia management model to improve medication adherence in comparable socio-economic contexts where human and financial resources were limited. Long-term cost-effectiveness assessment is needed.
*Cardiovascular diseases*													
17	Ajay et al. [32]	2014; Gongbujiang-da county, Linzhou county, Tibet Province	CVD	Community health workers(CHWs)	paid	With the smartphone-based electronic decision support, CHWs can provide 1. monthly follow-up care; 2. high-risk people identification; 3. referral; 4. therapeutic lifestyle advice (smoking cessation and salt reduction); 5. prescribe two drugs (blood pressure lowering drugs and aspirin)	1 year	Ended	Training on the intervention protocol, including key messages on targeted CVD lifestyle risks and medications	Initial training: not mentioned; refresh training: 1–3 months after intervention began	Lack of economic and healthcare resources	Supportive national guidelines and policies on CVD prevention and control	The first study to evaluate the effects of a simplified management program delivered by CHWs with the help of electronic decision support system on improving the health of high CVD risk patients. If effective, this intervention strategy can serve as a generalized model for similar settings
18	Yan et al. [33]	2014; Hebei, Liaoning, Ningxia, Shanxi and Shaanxi	CVD	Village Doctors	paid	1. Identified high-risk individuals by screening all patients in the village clinics. 2. contacted patients with existing diseases or potentially at high risk to maximize screening; 3. measured blood pressure; 4. provided lifestyle modification advice; 5. monitored acute symptoms or early signs of clinical events on monthly follow-up; 4) referral	2 years	Ended	A technical package developed to guide VDs on how to screen, identify, treat, follow-up, and refer cardiovascular high-risk individuals during their routine services.	Two 1-day sessions:1 before intervention, and again 1 month after the initiation of intervention	N/A	1. Performance-based feedbacks and financial incentivespayment increased motivation; 2. interventions were designed to fit CVD management in resource-limited areas	This was the first cluster-randomized trial in the world to assess the population impacts of a high-risk strategy in prevention and control of CVD. The technical interventions used were all evidence-based and tailored for VDs.
19	Tian et al. [15]	2015; Gongbu-jiangda county, Linzhou county, Tibet Province	cardiovascular disease (CVD)	Community health workers (CHWs)	paid	With the smartphone-based electronic decision support, CHWs provided 1. monthly follow-up care; 2. high-risk-patient identification; 3. referral; 4. therapeutic lifestyle advice (smoking cessation and salt reduction); prescription of two drugs (blood pressure lowering drugs and Aspirin); 5. screening for new symptoms, diseases, and side effects; 6. blood pressure measurement	1 year	Ended	Training on the intervention protocol, including key knowledge and skill on targeted CVD lifestyle risk factors and medications.	Initial training: duration not mentioned; refresher training: 1–4 months after the intervention began	1. The duration of the intervention was too short to observe significant health behavioral change; 2. lack of resources in remote areas	1. Performance-based incentives.	The CHWs were capable of delivering CVD management program with smartphone-based decision support system in rural settings in China and India.
*NCD risk factors*													
20	Li et al. [34]	2016; Hebei, Liaoning, Shanxi and Shaanxi provinces and the Ningxia Autonomous Region	sodium reduction	Village doctor	paid	Worked with township health educators to 1. provide health education in forms of public lectures; 2. distribute promotional materials; 3. conduct education sessions with vascular high-risk population; 4. promote salt substitute	18 months	Ended	N/A	N/A	N/A	N/A	Population sodium intake was reduced by this intervention, through increased use of salt substitute. Larger effects could be achieved in rural China by a wholesale switch from salt to salt substitute to prevent stroke.

Annotations: N/A, no relevant information in the article. Abbreviations: BPHS, Basic Public Health Service; CBT, cognitive behavioral therapy; CHSC, Community Health Service Center; CHS, Community Health Station; CHWs, community health workers; CLs, community leaders; CVD, cardiovascular disease; EHR, electronic health record; FHPs, family health promoters; LEAN, lay health supporter, e-platform, award, and integration; LHSs, lay health supporters; mHealth, mobile health; NCDs, non-communicable diseases; NCMS, New Cooperative Medical Scheme; PHC, primary health care; PLs, peer leaders; PLSP, Peer Leader Support Program; T2DM, type-2 diabetes; VDs, village doctors; DOT, directly observed therapy; SMS, short messaging service; MHA, mental health assistant.

## Abbreviations

CHW	Community health worker
CVDs	Cardiovascular diseases
THC	Township Health lefts
NCMS	New Cooperative Medical Scheme
NCDs	Non-communicable diseases
RCT	Randomized control trial
TB	Tuberculosis
VDs	Village doctors
WHO	World Health Organization
